# Telerehabilitation during the COVID-19 pandemic, what are the determinants of satisfaction for chronic diseases? a retrospective study

**DOI:** 10.3389/fresc.2023.1108087

**Published:** 2023-01-26

**Authors:** Anne laure Roy, Aurélie Duruflé, Patrice Piette, Bastien Fraudet, Vincent Lofficial, Philippe Gallien

**Affiliations:** Physical Medicine and Rehabilitation, Pôle MPR Saint-Hélier, Rennes, France

**Keywords:** tele rehabilitation, chronic low back pain, acceptability, neurological desorder, satisfaction

## Abstract

**Background:**

During the Covid-19 health crisis, telerehabilitation provided a solution to ensure the continuity of care. Since then, it has been offered as an alternative to face-to-face rehabilitation in chronic conditions. Data measuring satisfaction are essential to adapt and increase the effectiveness of this type of programme.

**Aim and scope:**

This research focused on determining the most significant determinants of participant satisfaction in a telerehabilitation programme.

**Methods:**

We conducted a retrospective study by analysing the satisfaction questionnaire used from the start of the programme.

**Result:**

Two hundred and ten (210) participants completed the programme; 180 questionnaires were filled in and 175 analyzed of which 70 with chronic low back pain (CLBP), 59 for multiple sclerosis (MS) and 22 with parkinson's disease (PD). Satisfaction was high for all participants (scoring out of 10, mean = 8.22 sd = 1.53), but the determinants reported for the three main conditions involved in the programme differed. Main determinant was “benefice” for CLBP (*p* = 1.23e-05), “home exercises adapted” for MS (*p* = 0.000679) and “interest in staying at home” for PD (*p* = 1.84e-05).

**Conclusion:**

Depending on the context of the condition/disease, the drivers of satisfaction were not identical. Knowledge of these determinants will allow us to further improve the programme. However, some unresolved questions remain regarding the place of therapists, their role and the skills required for a successful telerehabilitation programme. Further studies are required to understand the impact.

## Introduction

For chronic disorders, neurological or musculoskeletal, failure to receive routine rehabilitative care has potential implications for disease progression as well as functional deterioration and psychological distress (1). For these patients,, the negative effects of social distancing may be even greater, as they require regular follow-up to minimize the impact of the disease (2). Exercise therapy has been defined as a series of movements designed to train or develop the body through routine practice or as a physical workout to promote good physical health. They may contain for upper or lower limb: muscle power training, task based training, endurance components or muscle power balance training (3, 4). The implementation of these treatments involves several health professionals, physiotherapists, occupational therapists, but also psychologists to monitor the impact of the disease on mental health. These programs must be adapted to the level of functional independence, fatigability or age.

In the context of SARS-CoV-2 infection and the spread of the COVID-19 pandemic, health services needed to adapt and prioritize the provision of safe care, thus limiting outpatient services. We therefore needed to design an innovative method to offer a rehabilitation programme and ensure the continuity of care (5, 6). Telerehabilitation, which is still uncommon, has emerged as a potential solution. Telehealth is ideal when caring for transmissible conditions thus reducing person-to-person contact (7). It allows therapists to: (1) maintain continuity of care by educating patients through remote consultations directly in their own environment, (2) conduct a physical evaluation and plan a targeted therapeutic exercise programme, and (3) monitor the progress of patients by providing ongoing feedback and follow-up (8–10).

Since all therapists are familiar with face-to-face treatment, several stumbling blocks remain for the roll-out of telerehabilitation programmes. In terms of disadvantages, a problem could be the loss of human contact, face-to-face interaction, with the therapist (11). In fact, it is recognised that the effectiveness of physiotherapy relies not only on direct interventions but also on other contextual factors intrinsic to the experience of patients at the rehabilitation center or practice (12). In addition, during the clinical examination, the inability to palpate the patient and use other tests as diagnostic tools could compromise the follow-up process and objective assessment of the condition of patient or result in a failure to recognise warning signs (9, 13). Equipment problems such as lack of rehabilitation tools (elastic bands, weights, devices) could limit the provision of care and reduce the range of therapeutic exercises (9). Moreover Safety is a major concern to remote physiotherapy, in particular because of the limited possibility of direct intervention by the operator (14). telemedicine encounters are more vulnerable to privacy and security risks (15).

The question of the effectiveness of telerehabilitation was therefore raised very early on and numerous studies have examined the issue of effectiveness (16–20). In the area of neurological rehabilitation, most studies report no difference between face-to-face and telerehabilitation in terms of balance, functionality and quality of life (21). In musculoskeletal areas and especially in chronic lower back pain, the published literature is heterogeneous and that digital intervention studies for low back pain are generally poorly described. The literature does not provide sufficient detail regarding target and participant populations, intervention components, and rationale for the wide variety of outcome measures used. This makes it difficult to get a clear overview of what might work best, for whom, and under what circumstances (22–24).

On 17 March 2020, the French population went into lockdown to combat the COVID-19 epidemic. This was followed by successive waves of lockdowns and lifting of restrictions that lasted until the summer of 2022. Healthcare teams decided to offer patients the option to continue their rehabilitation *via* synchronous remote consultations combined with self-rehabilitation exercises. The aim was to bring intensive, interdisciplinary and personalized rehabilitation care into the home of the patient. The question of acceptability and patient satisfaction was therefore raised in a context of restricted choice. Moving forwards, knowledge regarding the level of satisfaction and its determinants will be used to assess and adapt the programme. The aim of this study is to assess the level of satisfaction of participants, to highlight the variables predicting satisfaction levels and to make assumptions regarding possible predictors not measured in our survey.

## Method

### Switch from face-to-face to remote consultations in a health-emergency context

Based on the face-to-face day hospital model, the programme consisted of one 30-minute session, three days a week, over a period of four weeks. These individual or collective sessions were provided by at least three different types of professionals such as physiotherapists, occupational therapists and adapted physical education teachers. The goal was to recreate at home the rehabilitation care set up in a rehabilitation center: intervention of various professionals (physiotherapist, occupational therapist, sports educator) with a care program focused on each patient face to face. Patients with low back pain received 5 individual sessions and 4 group sessions per week. Patients with neurological diseases received 4 individual sessions and 5 group sessions. Thus, each patient had two or three sessions per day. The session duration was 30 min for individual and 45 min for group. Depending on the patient, other professionals were involved including psychologists, neuropsychologists, social workers, dieticians or speech therapists. At the end of the programme, each patient was reviewed by the prescribing doctor during a teleconsultation. Patients were grouped together according to their condition: chronic lower back pain or neurological disorder (multiple sclerosis, Parkinson's disease or others). Each group was composed of three patients. To perform the telerehabilitation, the therapist and patient each needed a computer or a tablet with a camera. A smartphone can be used but the size of the screen is inadequate for group sessions. We recommended to patients that they should have a mat on the floor for some exercises but this was not essential. The baseline assessment was used to set the goals of the program and to establish the kind of exercises to be included in the training program. A self-education handbook, co-designed with the patient and the therapists, included the selected exercises. The posture of the exercises was selected according to the patient's abilities and preferences (standing, sitting, lying on the bed or on the floor). For patients with low back pain, exercises focused on muscle strengthening, stretching, relaxation and reducing kinesiophobia. For patients with neurological disorders, the exercises were task-oriented, balance improvement, strengthening and stretching.

At each session, the E-Kermed videoconferencing software (a specific videoconferencing platform for the health sector developed for the Brittany region) sent the patient a connection link by email. No software needed to be installed. A connection test was carried out before each session to ensure to check the adequacy of the connection.

The content of the sessions depended on the targets determined with the patient at the beginning of the session. The rehabilitation specialists performed a personalized assessment using validated scales where possible (Oswestry for lower back pain patients, EMIF for multiple sclerosis patients). Daily utensils were very often used as rehabilitation equipment including chairs, stairs, water bottles instead of dumbbells, etc.

### Construction of an assessment grid

A questionnaire was quickly prepared to collect patients' satisfaction on their course of treatment.

**Figure 1:**
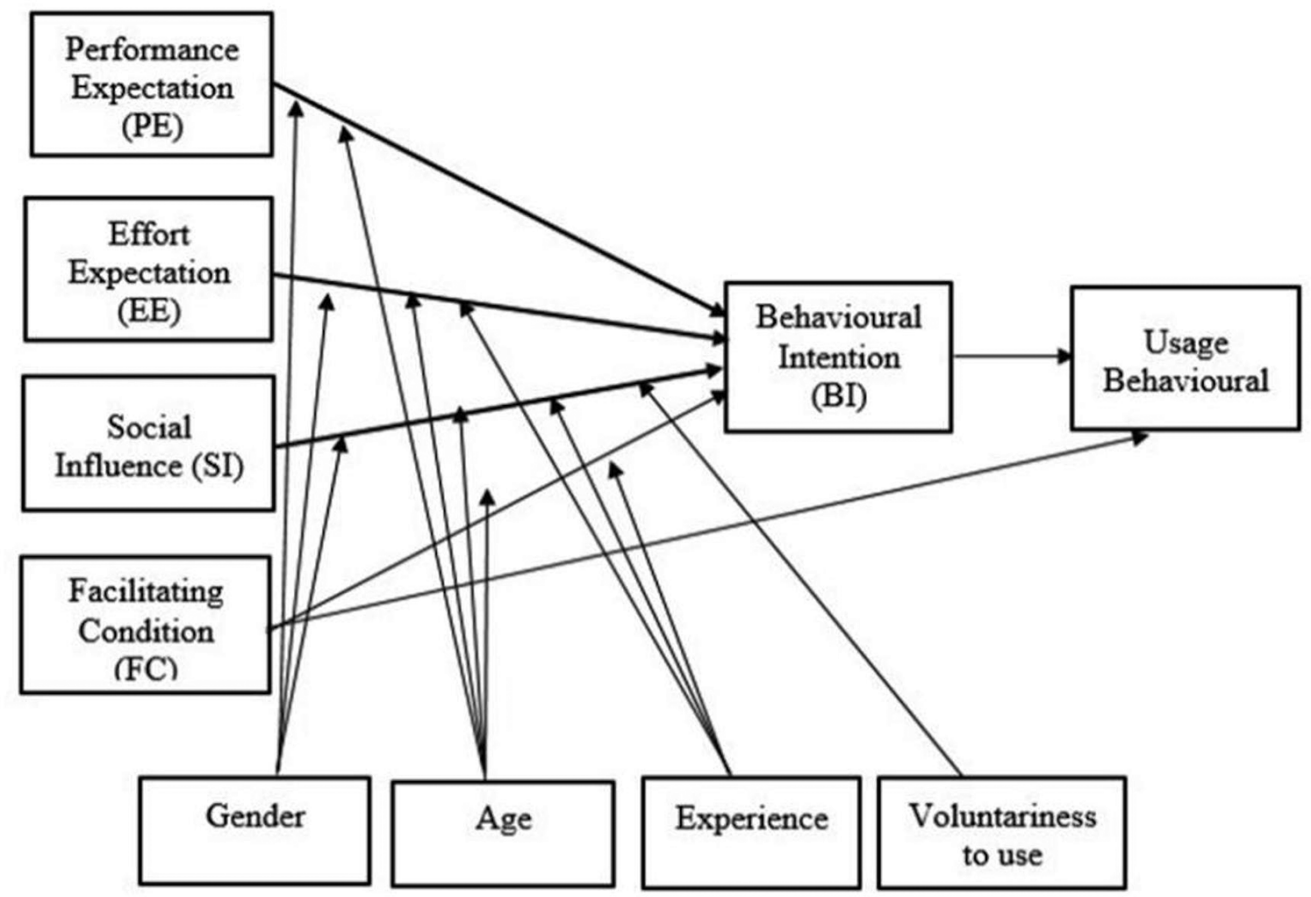
UTAUT model (26), available *via* license: CC bY-NC 4.0.

Satisfaction can be considered as the result of a judgment, of a cognitive process, of comparison between the expectations of the subject and the perceived reality (25). Then a series of questions based on the UTAUT were associated with satisfaction to study the determinants. The UTAUT model (Figure 1) uses four core determinants to determine users behavioral intention (BI) to use a technology: Performance expectancy (PE), effort expectancy (EE), social influence (SI) and facilitating conditions (FC) (26). These four major factors are defined as follows: Performance expectancy as “degree to which an individual believes that using the system will help him or her to attain gains in job performance”, effort expectancyas “degree of ease associated with use of the system”,social influence as “degree to which an individual perceives that important others believe he or she should use the new system” and facili-tating conditions as “degree to which an individual believes that an organizational and technical infrastructure exists to support use of the system”. Gender, age, experience, and voluntariness of use are moderating variables assumed to influence the four key variables on usage intention and behavior. The UTAUT theoretical model is derived from eight previous models of technology acceptance. The urgency required for setting up a digital day hospital meant no work was carried out on quality of the questionnaire. Moreover, it was not tested to assess its internal consistency. However, the research teams, familiar with UTAUT, quickly identified issues that could pose stumbling blocks to patient satisfaction (Table 1). Three main dimensions were included but social influence were not able to use due to the health restrictions.Indeed, the confinement did not leave the choice of the participants towards the alternative of face to face. Each question took the form of a Likert scale with parameters ranging from 0 to 10. The final question was an open-ended question about their rehabilitation programme. The questionnaire was upload on-line as a Google form with a link sent at the end of the treatment. Due to the non-secure nature of the questionnaire, responses were anonymous and no health data was requested, It was therefore impossible to analyze the results according to the moderating variables (gender, age)

**Table 1:** Presentation of the questionnaire – link between items and UTAUT dimensions.

Number	Description	UTAUT correspondence
Q1	How would you rate your overall satisfaction with the programme?	Effort expectancy
Q2	How would you rate your ease participation given the remote system set up?	Facilitating conditions
Q3	How would you rate the ease of connecting for the first time?	Facilitating conditions
Q4	How would you rate the ease of connecting the next times?	Facilitating conditions
Q5	How would you rate the value of doing the programme at home?	Performance expectancy
Q6	How do you rate the value of remote activities compared to the same activities face-to-face?	Performance expectancy
Q7	How would you rate the value of the inclusion of group activities in addition to individual sessions?	Performance expectancy
Q8	How would you rate the perceived physical intensity of this programme?	Performance expectancy
Q9	How do you rate the relevance of the self-rehabilitation exercises proposed?	Performance expectancy
Q10	Did you find the self-rehabilitation exercises appropriate for your needs?	Performance expectancy
Q11	Did you find the daily length of the self-rehabilitation exercises appropriate?	Performance expectancy
Q12	Did you find the difficulty of the self-rehabilitation exercises appropriate?	Performance expectancy
Q13	How would you rate your perceived benefit from this programme (your experience)?	Performance expectancy
Q14	Do you think you will continue with the self-rehabilitation exercises after this 4-week programme?	Intention to use
Q15	Would you recommend this day hospital programme to other people with the same needs?	Intention to use

### Method of recruiting participants

During the medical consultation, the doctor assessed the potential compliance of the patient, his/her wishes, ability to use the digital tools, connection, indication and need to set up self-exercises. Method of recruiting participants During the medical consultation, the doctor assessed the potential compliance of the patient, his/her wishes, ability to use the digital tools, connection, indication and need to set up self-exercise.For neurological disorders, the patients recruited had an EDSS of less than 5 for MS and a Hoehn and Yahr score of 3 or less for Parkinson's disease. For low back pain an initial bio-psycho-social assessment using the Dallas scale excludes patients with excessive psycho-behavioral impact.

### Data collection and analysis method

Data were analysed in R static version 4.01 *via* Rstudio. Data collection and analysis method Data were analyzed in R static version 4.01 *via* Rstudio*.* Cronbach's alpha coefficients was use *post hoc* to study internal consistency of questionnaire. Bayesian information criterion (BIC) was used as a criterion for model selection among a finite set of models (27). With the criteria resulting from BIC calculation, a linear model (estimated using Ordinary Least Squares, OLS) was perform to predict satisfaction.

## Result

### Participants

**Figure 2:**
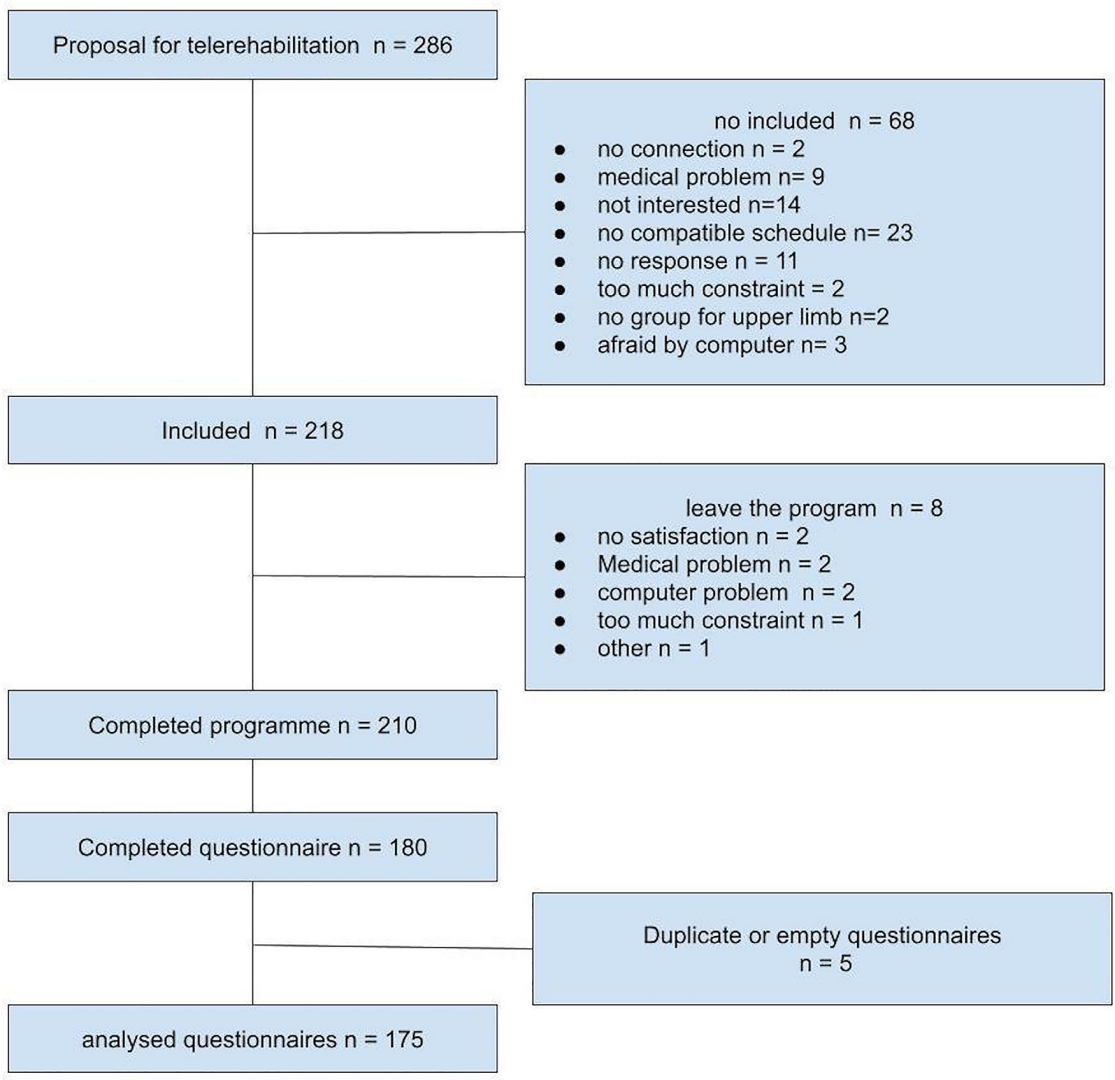
Flow chart: program integration of patients.

Between 17/04/2020 and 29/08/2022, 218 patients were enrolled in the programme, 210 completed the four weeks, 180 questionnaires were completed, five of which were duplicates. Overall, 175 usable questionnaires were analyzed (Figure 2). Out of a total of 218 patients enrolled, 109 were with low back pain (LBPGroupe), 56 with multiple sclerosis (MSGroupe), 34 with Parkinson‘s disease (PDGroupe) and 19 for other diseases. The majority of patients were female for LBPG (55.99%) and MSG (83.35%) and male for PDG (61.8%) The mean age and standard deviation was for LBPG 48.01 (11.00), MSG 49.48 (11.05) and for PDG 67.41 (7.16).

### Results of the questionnaire

Validation of the questionnaire was done post-hoc. Standardised Cronbach coefficient was calculated and is excellent 0.93 [0.91; 0.94]. Questionnaire show a very good internal consistency.

The results showed a good overall level of satisfaction (Table 2): mean(sd) = 8.22 (1.53). In the questions, the lowest score corresponded to the assessment of physical intensity, mean(sd) = 7.53 (1.54), the highest to self-rehabilitation exercises mean(sd) = 8.73 (1.86). The intention to recommend the programme was high, mean(sd) = 8.37 (1.82).

**Table 2:** Means and standard deviation of questions, score between 0 (very bad) and 10 (very good), see Table 1 for the definition of questions, *N* = 175.

Number	Mean (sd)
Q1	8.22 (1.53)
Q2	8.00 (1.80)
Q3	7.86 (2.39)
Q4	8.42 (1.87)
Q5	8.24 (1.74)
Q6	7.41 (2.01)
Q7	7.73 (2.28)
Q8	7.53 (1.54)
Q9	8.69 (1.30)
Q10	8.73 (1.86)
Q11	8.23 (1.42)
Q12	8.44 (1.52)
Q13	7.89 (1.97)
Q14	8.51 (1.50)
Q15	8.37 (1.82)

### Satisfaction analysis of the entire population

Post-hoc analyses were carried out on the prediction of satisfaction using the other pre-event questions. We used ANOVA to compare differences of means among diseases by examining the amount of variability between the samples relative to the amount of variability within the samples. No difference of satisfaction was observed.

**Figure 3:**
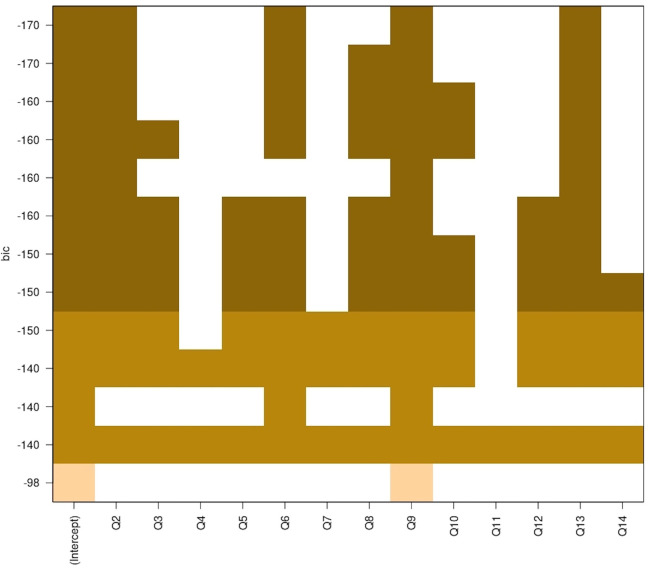
Bayesian criterion information, likelihood of predictive satisfaction combinations.

A Bayesian information criterion (BIC) was performed to select the best predictive criterion (Figure 3). Then, a linear regression was performed to model the relationship between “satisfaction” (dependent variable) and the other questions (explanatory variable).

Thus, several variables were not considered to be predictive of satisfaction (Table 3), i.e.,: the length of the sessions *p* value = 0.61; the ease of the first connection *p* value = 0.20, the ease of the connections *p* value = 0.83, group activities *p* value = 0.16.

**Table 3:** Linear model of predictive values on satisfaction, pooled sample.

Coefficients	Estimate	Std. Error	*t* value	Pr (>|*t*|)
(Intercept)	0.61842	0.48228	1.282	0.20155
Facilite	0.2041	0.04706	4.337	2.52e-05 ***
Interetprogramme	0.11166	0.06235	1.791	0.07515.
Rapportpresentiel	0.12107	0.05687	2.129	0.03475 *
Intesitephysique	0.1064	0.05438	1.957	0.05209.
Autoreeducationadapt	0.22934	0.06579	3.486	0.00063 ***
Benefice	0.17048	0.04951	3.443	0.00073 ***
Multiple R-squared: 0.6756, Adjusted R-squared: 0.6638, *p*-value: < 2.2e-16				

Signif. codes: 0 “***” 0.001 “**” 0.01 “*” 0.05 “.” 0.1 “ ” 1, Facile = facilitation of use, Intereteduprogramme = interest to be at home, rapportpresentiel = interest of programme and exercice compare to presentiel, intensitephysique = physical intensity adapted, autoreeducationadaptée = home exercices adapted, benefice = effect size feeling, *N* = 175.

After refining the model by eliminating the non-predictive variables, the regression model retained three major variables predicting satisfaction (Table 2): ease of use (*p* value = 2.52e-05), adapted self-rehabilitation (*p* value = 0.0006) and the perceived benefit (*p* value = 0.00073). Plus three minor variables: relationship to face-to-face (*p* value = 0.03475), interest of the programme of being at home (*p* value = 0.07515) and physical intensity (*p* value = 0.05209).

### Analysis of satisfaction by disease

A variable elimination procedure was conducted to refine the linear regression model by condition. Three groups sufficiently represented in the sample were selected (Table 4): chronic lower back pain (LBP, *n* = 70), Parkinson's disease patients (PARK, *n* = 22) and multiple sclerosis patients (MS, *n* = 59). The refined models showed that ease of use was an item shared by all patients. However, for the performance expectancy dimension, the predictive points for satisfaction diverged. The LBP group emphasized the perceived benefit of the programme (*p* value = 1.23e-05), the MS group the relevance of the self-rehabilitation exercises (*p* value = 0.00068) and the PARK group the benefit offered by the programme of being at home (*p* value = 1.84e-05) and the exercises relative to their expectations. The variance explained was very high in the PARK group (Adjusted R-squared: 0.7914, *p*-value: 1.322e-07), moderate for the MS group (Adjusted R-squared: 0.5103 *p*-value: 3.072e-09, and high for the group LBP (Adjusted R-squared: 0.66, *p*-value: 7.659e-15).

**Table 4:** Linear model of predictive values on satisfaction.

Coefficients	Estimate	Std. Error	*t* value	Pr (>|*t*|)
* LBP n* = 70 (Intercept)	0.56318	0.77203	0.729	0.46837
Facilite	0.26235	0.091	2.883	0.005359 **
Interetprogramme	0.32493	0.08209	3.958	0.000192 ***
Faciliteconnection1	−0.1353	0.05689	−2.378	0.020416 *
Intesitephysique	0.20706	0.09199	2.251	0.027842 *
Benefice	0.29179	0.06154	4.742	1.23e-05 ***
Multiple R-squared: 0.6846, Adjusted R-squared: 0.66 *p*-value: 7.659e-15				
MS *n* = 59 (Intercept)	0.2360	1.0877	0.217	0.829002
Facilite	0.3214	0.1026	3.133	0.002772 **
Autoreeducationadapte	0.4260	0.1183	3.602	0.000679 ***
Benefice	0.2114	0.1009	2.096	0.040652 *
Multiple R-squared: 0.5356, Adjusted R-squared: 0.5103 *p*-value: 3.072e-09				
*PARK: n* = 22 (Intercept)	1.75680	0.68099	2.580	0.01836 *
Facilite	0.29153	0.07555	3.859	0.00106 **
Interetprogramme	0.53394	0.09424	5.666	1.84e-05 ***
Multiple R-squared: 0.8112, Adjusted R-squared: 0.7914 *p*-value: 1.322e-07				

LBP, low back pain, MS, multiple sclerosis, PARK, Parkinson's disease. Signif. Codes: 0 “***” 0.001 “**” 0.01 “*” 0.05 “.” 0.1 “ ” 1, Facile = facilitation of use, Intereteduprogramme =, rapportpresentiel = interest of programme and exercice compare to face to face, intensitephysique = physical intensity adapted, autoreeducationadaptée = home exercices adapted, benefice = effect size feeling.

### Analysis of the open-ended text

91 participants responded to the open-text question amounting to a total of 5,168 words. The open-ended text was analyzed using Rstudio, Text Mining Package V 0.7.9 and Wordcloud V 2.6. The analysis of word occurrences listed the 17 most frequently used words (Table 5), represented as a word cloud.

**Table 5:** Analysis of the occurrence of vocabulary used in the open-ended text, the 17 most frequent words Physiotherapists and occupational therapists were grouped together under the term therapist.

Word	Freq
très (*very*)	47
séance(s) (session(s)	41
bien, bonne (*well*)	41
programme (*programme*)	36
plus (*more*)	28
exercices (exercises)	26
merci (*thank*)	26
intervenants (*therapists*)	24
tout (*all*)	21
temps (*time*)	20
être (*to be*)	15
faire (*doing*)	14
moins (*less*)	13
rééducation (*rehabilitation*)	13
travail (*work*)	11
semaines (*week*)	10

Under the term “therapist”, we grouped together the different names of therapists, sometimes by first name, last name, or simply named as therapists (physiotherapist, occupational therapist, etc.).

## Discussion

The results corroborate the high level of satisfaction with telerehabilitation in chronic conditions (28–33). These results corroborate that tele-rehabilitation not only provides access to rehabilitation programs, but also reinforces participant-centered treatment, adherence to rehabilitation and lifestyle changes over time (34). A systematic review (*n* = 44 studies) investigating the association between telehealth and patient satisfaction found that patients were satisfied when using telehealth programmes, as they produce similar outcomes, are easy to use, improve communication and reduce travel time (35). Our study provided additional information on the key predictors of satisfaction and showed that not all patients have the same sensitivity to the features of the devices and that these differences were related to the condition of the patient. These results are consistent with the feedback received from patients during medical consultations. The population of MS patients are younger and place a high value on self-rehabilitation in unsupervised conditions. Parkinson's patients find it very hard to travel and remote exercising solutions offer them an interesting alternative. The LBP group, unlike the other two, do not suffer from a progressive illness and their condition improves with treatment, meaning that they were more aware of the perceived benefit.

However, the explained variance study reported the presence of other predictive factors not revealed by the questionnaire, especially for the MS group, whose explained variance was only 0.51.

### Place and role of therapists

The analysis of the open-ended text reveals a dimension not explored by the questionnaire, i.e., the place of therapists. This word was placed in eighth position among the most commonly used words in the analysis of the open-ended text. Compared to studies on e-learning, therapists may have a significant impact on the success of telerehabilitation. The predictive criteria mentioned in e-learning are: the position of the therapist in terms of distance learning (36, 37), their skills in the area of new communication techniques (38), their capacity for appropriate feedback (38, 39) and the quality of their oral expression (40). These criteria could be transferable to telerehabilitation, as they were associated with other dimensions in the questionnaire such as ease of use, motivation of the subjects (41). This key role of therapists in success has been discussed in studies of other conditions such as COPD (29).

### Adverse effects

The teams reported three minor and non-serious falls out of a total of 1,744 supervised sessions. This low rate probably related to the selection criteria and the caution of therapists in terms of the difficulty of the exercises.

### Cost

No costing was carried out, however, given the location of the participants, it was calculated that approximately 73,000 transport kilometers were saved. Moreover, no specific investment was required. Some patients took the opportunity to invest in standard communication equipment, but the vast majority already had a good internet connection and a screen. In terms of equipment, everyday objects available in the home were used. This programme therefore provides economic arguments in favor of its widespread roll-out.

## Limitation

Some descriptive information and health data are missing that would have allowed work on subgroups.

From a sampling perspective, while the lower back pain and MS groups were sufficiently large, the sample size of the PARK group was small.

Finally, there was a sample selection bias. The programme was based on voluntary participation and the presence of operational connection equipment.

## Conclusion

The high level of satisfaction of patients is consistent with the scientific literature. Various determinants were highlighted in our study according to the condition involved. The rarity of adverse events and the low economic set-up cost are positive drivers for the widespread roll-out of such programmes. However, some questions remain, in particular about the role of therapists and their associated skills in the success of these programmes. In addition, long-term follow-up is required to study patient adaptation and acceptance in a normalised health context.

## Data Availability

The data analyzed in this study is subject to the following licenses/restrictions: Datasets are available on request: The raw data supporting the conclusions of this article will be made available by the authors. Requests to access these datasets should be directed to patrice.piette@pole-sthelier.com.
